# Aptamer Functionalized Lipid Multilayer Gratings for Label-Free Analyte Detection

**DOI:** 10.3390/nano10122433

**Published:** 2020-12-05

**Authors:** Plengchart Prommapan, Nermina Brljak, Troy W. Lowry, David Van Winkle, Steven Lenhert

**Affiliations:** 1 Department of Physics, Florida State University, Tallahassee, FL 32306, USA; pp11d@my.fsu.edu (P.P.); twl10@my.fsu.edu (T.W.L.); dvanwinkle@fsu.edu (D.V.W.); 2 Department of Chemistry, Florida State University, Tallahassee, FL 32306, USA; n.brljak@miami.edu; 3 Department of Biological Science and Integrative Nanoscience Institute, Florida State University, 77 Chieftan Way, Tallahassee, FL 32306, USA

**Keywords:** biosensor, aptamer, diagnostics, grating, transducer, vesicle, multiplexed, photonics, array, nanofabrication

## Abstract

Lipid multilayer gratings are promising optical biosensor elements that are capable of transducing analyte binding events into changes in an optical signal. Unlike solid state transducers, reagents related to molecular recognition and signal amplification can be incorporated into the lipid grating ink volume prior to fabrication. Here we describe a strategy for functionalizing lipid multilayer gratings with a DNA aptamer for the protein thrombin that allows label-free analyte detection. A double cholesterol-tagged, double-stranded DNA linker was used to attach the aptamer to the lipid gratings. This approach was found to be sufficient for binding fluorescently labeled thrombin to lipid multilayers with micrometer-scale thickness. In order to achieve label-free detection with the sub-100 nm-thick lipid multilayer grating lines, the binding affinity was improved by varying the lipid composition. A colorimetric image analysis of the light diffracted from the gratings using a color camera was then used to identify the grating nanostructures that lead to an optimal signal. Lipid composition and multilayer thickness were found to be critical parameters for the signal transduction from the aptamer functionalized lipid multilayer gratings.

## 1. Introduction

Miniaturized biosensors are promising components for portable diagnostics and environmental monitoring through the detection of multiple analytes from a single (~15 µL) droplet of liquid [1,2]. A critical step in a biosensing process is the transduction event, where an analyte interacts with the sensor to produce a signal that can be detected. The transduction step most often involves analyte binding to a transducer which triggers an electronic or optical signal. A variety of transduction elements have been investigated, including electrodes [3,4,5,6], semiconductor nanowires [7,8,9], plasmonic surfaces [10,11], nanoparticles [12,13,14], cantilevers [15,16], quartz crystals [17,18], optical fibers [19,20], liquid crystals [21,22,23], photonic materials [11], and diffraction gratings [24].

Lipid multilayer gratings are a promising type of transducer, based on monitoring changes in diffraction intensities upon binding [25]. When analytes bind to the gratings and interact with reagents contained by the fluid lipid volumes, nanoscopic shape changes lead to changes in optical diffraction. Monitoring the pattern of reaction can be used to detect analytes that are not necessarily specific for any one chemical functionality using an optical nose approach [26]. Lipid multilayer gratings composed of the model phospholipid 1,2-dioleoyl-*sn*-glycero-3-phosphocholine (DOPC) as well as DOPC mixed with the cationic lipid analog 1,2-dioleoyl-3-trimethylammonium-propane (DOTAP) have been shown to be sensitive to vapors in the air and for measurements of pH or membrane binding protein kinetics under water [25,26,27].

The biofunctionalization of transducer elements with high spatial resolution is a limiting factor in multiplexed detection, i.e., detecting multiple analytes from the same sample [9,28,29,30]. Covalent linkage of an antibody or aptamer to a transducer surface is a well-established approach to biosensor functionalization [31,32,33]. Antibodies are highly specific and well characterized biological macromolecules that can demonstrate the highest selectivity [34]. The binding affinity and selectivity of both aptamers and antibodies depends on their environment [33,35]. While antibody denaturation is often irreversible, aptamers tend to be less sensitive to dehydration than antibodies, and can re-hybridize to reestablish their conformation if denatured during the sensor fabrication process [36]. Antibodies are also typically limited to the detection of large molecules or aggregates due to the requirement for two antibodies to cluster and bind to an antigen during their development [37]. Aptamers have binding affinities comparable to antibodies and can bind specifically to various targets, ranging from metal ions [38] to small molecules [39] and to larger entities such as cells [40,41].

Lipid gratings provide a convenient approach to the integration of different biofunctionalities because they do not rely on covalent modifications, but can have the functional biomolecule included in a mixture prior to fabrication. Lipid multilayer gratings have been fabricated by dip-pen nanolithography and nanointaglio printing [25,42,43,44]. Nanointaglio is the process used here, which involves arraying lipid-based inks onto a topographical grating, and then transferring the ink from the recesses of the stamp onto a flat surface, leaving a diffraction grating formed by the stamp [43,44]. Different materials can be readily integrated onto the nanointaglio stamp for the integration of different inks onto the same surface [44]. The multilayer thickness can be controlled in this process, either by stamp geometry or by controlling the amount of ink on the stamp [43,44].

Aptamers can be associated with lipids by conjugating them with hydrophobic molecules such as porphyrin [45] or cholesterol [46] that can partition into the hydrophobic lipid phase. In particular, the combination of two cholesterols on a double-stranded DNA molecule has been shown to lead to an irreversible coupling between functionalized DNA and lipid bilayer membranes [46]. This architecture has been used to study vesicle fusion [47,48]. Aptamer functionalized liposomes have been used for targeted drug delivery [49,50,51].

Here, we use this double-cholesterol tagged linker to functionalize lipid multilayer gratings with aptamers. We chose the DNA aptamer that binds to the protein thrombin as a model because it is one of the most well characterized aptamer-analyte combinations. In the absence of thrombin, the aptamer is in a loose random coil structure [52]. However, in the presence of thrombin the aptamer forms a G-quartet binding to the thrombin [52,53,54,55]. The 15-mer single-stranded DNA aptamer (5′-GGTTGGTGTGGTTGG-3′) binds to the fibrinogen recognition sites with a K_d_ of 26 nM [56,57]. Previous research has studied the influence of the aptamer immobilization to solid surfaces and optimized binding assay conditions [58,59]. Here, we combine the advantages of fast, inexpensive, and scalable lipid multilayer nanoarrays and the high stability of aptamers over antibodies to demonstrate this biosensing concept.

**Figure 1:**
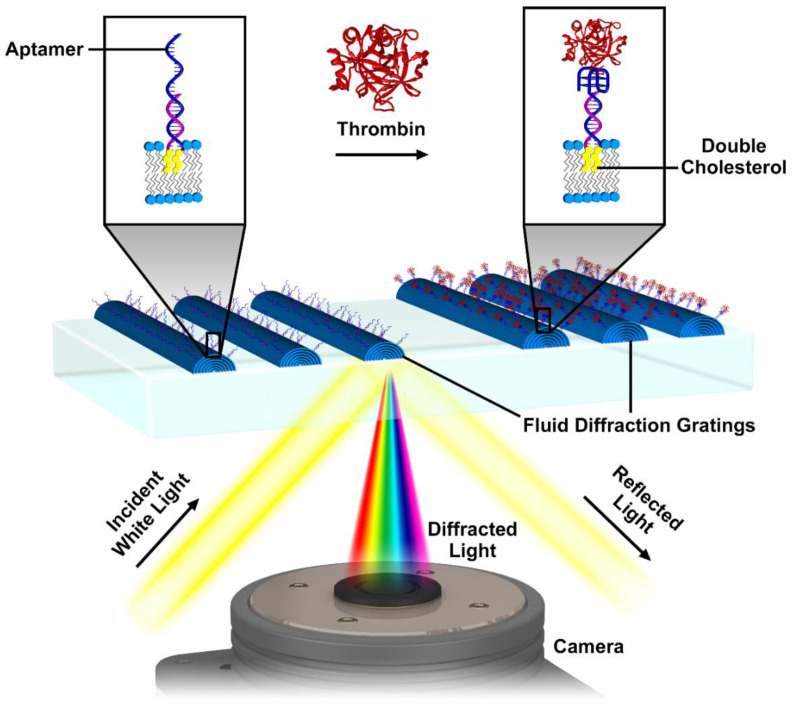
Schematic illustration of the use of aptamers to functionalize lipid multilayer gratings for selective analyte detection. Aptamers are incorporated into lipid multilayer gratings using a double cholesterol-tagged, double-stranded DNA linker. The gratings are illuminated at an angle, and the diffracted light is detected using a color camera. Exposure to thrombin and binding events can be detected by monitoring the intensity of diffracted light, and the signal is amplified by nanostructural changes in the lipid grating elements.

A schematic of the aptamer functionalized lipid multilayer gratings used for the detection of thrombin is shown in Figure 1. A double cholesterol-tagged, double-stranded DNA linker is used to attach the aptamer to the phospholipid grating elements. Cholesterol-modified DNA is readily available, and the DNA modification can be accomplished by hybridization between a 15-mer DNA and a 30-mer DNA. The modified aptamers are incorporated into the lipid grating ink prior to fabrication. After the exposure of the grating to the target of the aptamer (thrombin), a nanomechanical change in the shape of lipid gratings occurs which can be detected by optical diffraction. We understand this transduction mechanism by thinking of the fluid grating elements as nanoscopic droplets. When molecules bind to the lipid-water interface of those droplets, the surface tension changes, which changes the droplet shape and height, which in turn affects the diffraction efficiency.

## 2. Materials and Methods

### 2.1. Materials

Both 5′ and 3′ cholesterol-modified, single-stranded DNA oligomers were obtained from Eurofins (Lancaster, PA, USA). The lipids, 1,2-dioleoyl-*sn*-glycero-3-phosphocholine (DOPC) and 1,2-dioleoyl-3-trimethylammonium-propane (DOTAP), were obtained from Avanti Polar Lipids (Alabaster, AL, USA). The fluorescently labeled phospholipid, Marina Blue^®^ DHPE, was purchased from Life technologies (Eugene, OR, USA). The thrombin was purchased from Haematologic Technologies (Essex Junction, VT, USA) and then diluted to 2 mg/mL in PBS buffer pH 7.2 for fluorescent labeling. For the fluorescently labeled thrombin, the Pierce Rhodamine antibody labeling kit including the purification resin was purchased from Thermo Fisher Scientific (Waltham, MA, USA).

### 2.2. Fluorescent Thrombin Preparation

The thrombin solution was passed through a 0.2 µm syringe filter and then fluorescently labeled using the rhodamine labeling kit. After removing excess dyes by the columns and purification resin, the thrombin solution was filtered with a 0.2 µm filter. The concentration of labeled thrombin was 0.46 mg/mL, as determined by a Thermo Fisher Scientific NanoDrop 2000 Spectrophotometer (Waltham, MA, USA).

### 2.3. Aptamer Functionalized Vesicle Preparation

The double-stranded DNA with overhangs was formed by hybridization of a 30-mer DNA and 15-mer DNA with cholesterol modification by standard annealing. The 15-mer DNA sequence is Cholesterol TEG + 5′ CCC GAT CTC CTG CTT 3′. The 30-mer DNA sequence is 5′ GGT TGG TGT GGT TGG AAG CAG GAG ATC CCC 3′ + Cholesterol with Triethyleneglycol (TEG), whereas the overhang GGT TGG TGT GGT TGG is the thrombin aptamer [60]. On the other hand, the DNA with sequence 5′ TAG TTG TGA CGT ACA AAG CAG GAG ATC CCC 3 was used as the control DNA. To anneal the modified DNA, both the 30-mer and the 15-mer oligonucleotides were resuspended in the annealing buffer consisting of 10 mM Tris, pH 7.5, 50 mM NaCl, 1 mM EDTA. Equal volumes of both oligos were mixed at equimolar concentration. Then, the mixture was placed in a hot water bath to reach the temperature 95 °C. Finally, the mixture was allowed to cool down slowly to room temperature for 3 h. The concentration of stock solutions of DNA was 100 µM. Liposomes with Marina Blue DHPE 3 mol% were prepared by evaporation of chloroform under nitrogen gas, then deionized water was added followed by 10 min ultrasonication. The concentration of liposomes was 12.5 mg/mL. The annealed double-cholesterol DNA in the Tris buffer was then incubated with lipid liposomes for 30 min. The molar ratio of cholesterol-DNA to liposomes was 0.5 mol%.

### 2.4. Grating Fabrication

The mixture of cholesterol-based DNA and lipids was printed as lipid multilayer gratings by the nanointaglio method [44]. To fabricate these gratings, the functionalized liposome solution was deposited onto a polydimethylsiloxane (PDMS) grating stamp, and the solvent was evaporated. Finally, the lipid gratings were printed onto a Polymethylmethacrylate (PMMA) slide (HESA^®^ Glas HT, Notz Plastics, Brugg, Switzerland). For stability upon immersion, the lipid gratings were kept in a nitrogen glovebox for at least 2 days before the initial immersion in the buffer with 1% BSA [44]. The buffer used for grating immersion and aptamer binding was the selection buffer (20 mM Tris-acetate, pH 7.4, 140 mM NaCl, 5 mM KCl, 1 mM CaCl_2_, 1 mM MgCl_2_) used to identify the aptamer sequence, with an addition of 1% BSA [38]. The gratings were left to incubate in the re-hybridization buffer for 30 min at ambient room temperature.

### 2.5. Data Aquisition and Image Analysis

Fluorescence micrographs and sensorgrams were obtained on a Ti-E epifluorescence inverted microscope (Nikon Instruments, Melville, NY, USA) fitted with a Retiga SRV (QImaging, Ostrava, Czech Republic) CCD camera equipped with red, green and blue (RGB) filters, 1.4 MP, Peltier cooled to −45 °C). Marina blue fluorescence was detected with the Nikon filter set UV-2E/C for excitation at 340–380 nanometers and emission at 435–485 nanometers. Rhodamine fluorescence was detected with the Nikon filter set B-2E/C for excitation at 528–553 nanometers and emission at 590–650 nanometers. For imaging the diffracted light, a fiber-optic white light source (Eco Light 150, MK Photonics, Albuquerque, NM, USA) was positioned to illuminate the sample at an angle of about 50° from perpendicular.

## 3. Results and Discussion

**Figure 2:**
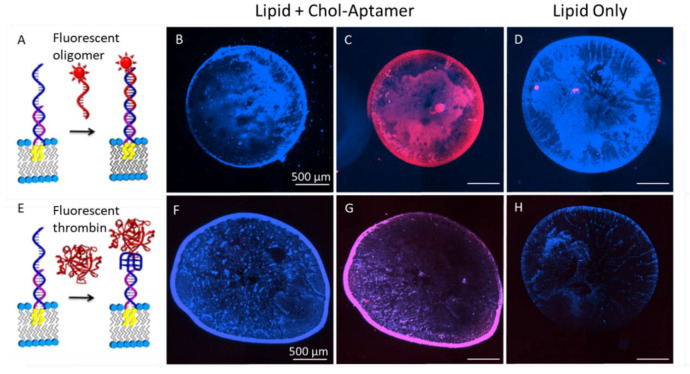
Aptamer functionalization of thick lipid droplets and binding of fluorescently labeled cDNA and aptamers. The lipids were fluorescently labeled blue in this experiment, and the analytes were labeled with a red fluorophore. The images show fluorescence overlays of both red and blue channels. (**A**) Schematic illustration of the use of a fluorescently labeled oligomer to detect the aptamer sequence. (**B**,**C**) Fluorescence images of a large (millimeter scale) aptamer functionalized lipid multilayer spot before (**B**) and after (**C**) exposure to the complementary oligomer fluoresced in red. (**D**) A lipid spot after exposure to the oligomer as a negative control. (**E**–**H**) The same experiment was carried, but with the oligomer replaced with the target of the aptamer; in this case the fluorescently labeled protein was thrombin.

We first tested the suitability of our strategy to incorporate aptamer functionality into lipid droplets using thick lipid multilayers, as shown in Figure 2. Aqueous solutions of aptamer functionalized liposomes composed of the phospholipid DOPC were spotted onto PMMA surfaces with a pipette and then dried in a vacuum, leaving multilayers with an average thickness of approximately 1.0 μm. DOPC was chosen as the lipid, as it is a well-characterized model phospholipid and it has been previously used to make lipid multilayer gratings [25,26,28,44]. Lipids were doped with a blue fluorescent label, and analytes were fluorescently labeled with a red fluorescent label for detection by fluorescence microscopy. Lipid multilayers were immersed into an aqueous solution and exposed to analyte for 30 min before washing to remove excess analyte. Aptamer sequences were detected in the lipid multilayers by binding fluorescently labeled complementary DNA (cDNA), as shown in Figure 2A–D. This indicated the presence of the aptamer sequence, and its accessibility to the molecules in the solution. Importantly, it can be observed that thicker regions bound more analyte than thinner regions, which means the analyte is likely partitioning into the lipid multilayer volumes. The edges of the spots are thicker than the center, as indicated by the brighter blue fluorescence [61]. The apparently larger amount of material at the edges of the spots is consistent with the coffee ring effect for drying of solutes from evaporated droplets [62]. The bound DNA fluorescence signal appeared unchanged after several washing cycles, indicating stable binding. A control that did not contain the aptamer sequence indicated that only functionalized lipids bound the cDNA. The result confirmed that the DNA was successfully embedded within the lipid multilayer spots, and its functionality survived the drying and rehydrating process.

We then tested the ability of these micrometer-thick aptamer functionalized multilayers to bind the aptamer target thrombin (Figure 2E–H). Since the aptamer-analyte binding affinity is known to depend on the environment (e.g., buffer, pH, local environment near a surface, etc.) [63], it is important for us to test the binding affinity of the aptamer for thrombin when attached to lipid multilayers. Fluorescently labeled thrombin was found to be successfully bound to the aptamer on the lipid spots, as shown by the red areas in Figure 2G. These results show that the aptamer qualitatively functions as anticipated when embedded in lipid spots even after dehydration and rehydration during the fabrication process. It is worth noting the differences in tone and intensity of the red areas in Figure 2C,G. This could be due either to a thrombin’s lower binding as compared to the double-stranded DNA or to differences in the fluorescent labels used for the complementary DNA and thrombin. The emission wavelength for the labeled thrombin is 575 nm with the extinction coefficient 80,000/mol⋅cm, while it is 603 nm with 11,600/mol⋅cm for the complementary DNA.

It was also observed that the thicker lipid multilayers around the spot edges bound more thrombin than the thinner multilayers, indicating either a partitioning of the analyte into the multilayer volumes or a larger number of binding sites available on the surface of the thicker multilayers. Although it is surprising that large molecules can incorporate into the volume of these lipid multilayer structures, due to the impermeability of lipid bilayers to water soluble proteins, this observation is in agreement with the His-GFP protein’s binding and intercalating into thick NTA-Ni lipids [64].

**Figure 3:**
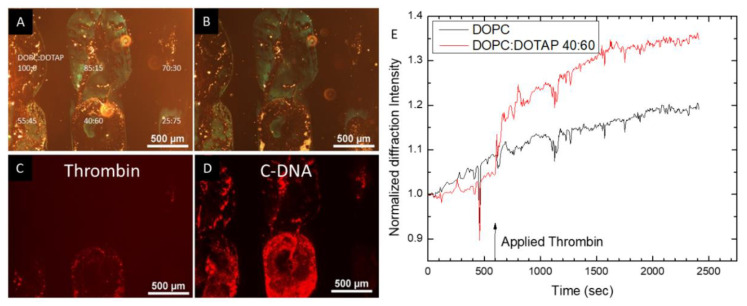
Lipid formulation was found to be critical to analyte binding to the gratings and detection by the monitoring of diffraction. (**A**) diffraction image of an array of spots of varying DOPC:DOTAP ratios. (**B**) diffraction image of the sample in A after exposure to fluorescently labeled thrombin. (**C**) Fluorescence micrograph of the sample after exposure to fluorescently labeled thrombin. (**D**) Fluorescence micrograph of the sample in (**C**) after exposure to fluorescently labeled cDNA for the aptamer sequence. (**E**) Sensorgram showing the optical response of the gratings upon exposure to analyte. There is a signal detected in the DOPC:DOTAP 40:60 gratings, but not in the DOPC control.

Next, we used the lipid inks to fabricate lipid multilayer gratings using nanointaglio printing. However, in comparison to the larger, millimeter-sized droplets, we were not able to detect the binding of analytes to these gratings either by fluorescence microscopy or by monitoring the diffraction. This is not surprising, since the multilayer thickness of lipid multilayer gratings is generally not higher than 1/10 of the line width [27]. Grating lines in this case are 300 nm, and grating heights are therefore expected to be less than 30 nm, which is more than 30 times thinner than the micrometer-thick multilayers from Figure 2. We hypothesized that the signal could be improved by varying the lipid composition [65]. Since cationic lipids are commonly used to incorporate nucleic acids into liposomes [66], we tested the effect of the cationic lipid DOTAP (Figure 3). The molar ratio of DOPC:DOTAP was varied, and gratings as well as thicker multilayers were printed from areas of the nanointaglio stamp that had excess ink. An image of an array of six different lipid compositions is shown in Figure 3A. Diffraction images such as these were taken with incident white light at such an angle that the gratings showed a green color in the image. Figure 3B shows a diffraction image of the pattern after exposure to the fluorescently labeled thrombin analyte. Figure 3C shows a fluorescent image of the same area. From experiments such as this we found that a DOPC:DOTAP molar ratio of 60:40 was optimal for the binding of thrombin. Figure 3D shows a fluorescence image of the same sample after exposure to fluorescently labeled cDNA, indicating the most binding to the 60:40 mixture. The analysis of the grating response to analyte binding also showed a detectable signal compared to DOPC controls, as shown in Figure 3E. The mechanism of improvement from the DOTAP could be due to more aptamer being present in the gratings, a higher binding affinity, and/or a higher grating line thickness.

**Figure 4:**
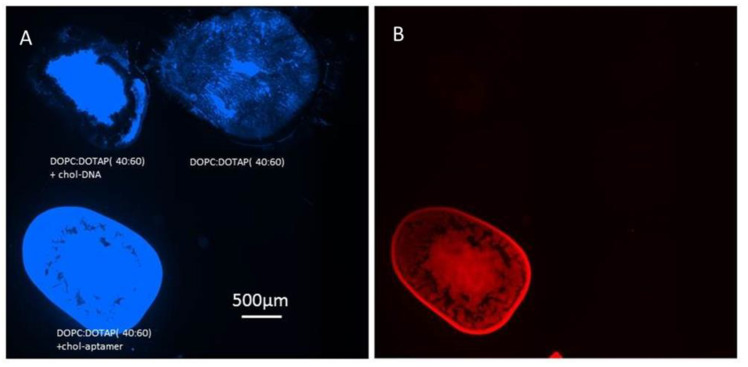
Control experiment to test if the binding to the DOPC:DOTAP 40:60 formulation was due to the aptamer or to electrostatic interactions. (**A**) Blue fluorescent image of an array of micrometer-thick multilayer spots composed of three different lipid compositions, DOPC:DOTAP 40:60 with a non-aptamer control DNA sequence, DOPC:DOTAP alone, and DOPC:DOTAP 40:60 with the aptamer sequence. The lipids were labeled blue. Panel (**B**) shows a fluorescence micrograph of the same array after exposure to fluorescently labeled thrombin, showing analyte binding only to the aptamer functionalized lipids.

Additionally, the lipids consisting of mixtures of DOTAP and DOPC were used to test the possibility of false positive signals caused by non-specific binding. For instance, since DOTAP should be positively charged, and DNA negatively charged at physiological pH, one might expect the DNA to bind to the DOTAP containing spots through charge interactions. As the non-specific blocking protein BSA is included in the buffers here, it is reasonable to assume that this protein may block non-specific absorption. To test for non-specific binding as well as the selectivity of our functionalized multilayer lipids, a non-aptamer sequence of DNA was introduced (Figure 4). It was confirmed that the red-labeled thrombin was bound only to lipid spots functionalized with the aptamer, and not to the cationic lipid-containing spot or the spot functionalized with non-aptamer sequences. This confirmed that our sensing system detected thrombin, and it is interesting that the response was quick, showing a signal within a minute of analyte addition.

Although we could now detect binding to the gratings by monitoring diffraction, the signal was very noisy and not always reproducible. We therefore sought to improve the signal to noise ratio by identifying which regions of the gratings produced the strongest signal. This is consistent with previous experiments relating to the binding of proteins to lipid multilayer gratings, in which we found that a minimum multilayer thickness was necessary for the gratings to function as a transducer for membrane-binding proteins [28]. Since we were monitoring the diffraction using a digital camera, each pixel in a time lapse recording represented a different area of the sensor. Heterogeneities in the grating structure due to different amounts of ink on different parts of the nanointaglio stamp could therefore be exploited in order to test hypotheses about how the lipid nanostructure relates to the function as an optical transducer.

**Figure 5:**
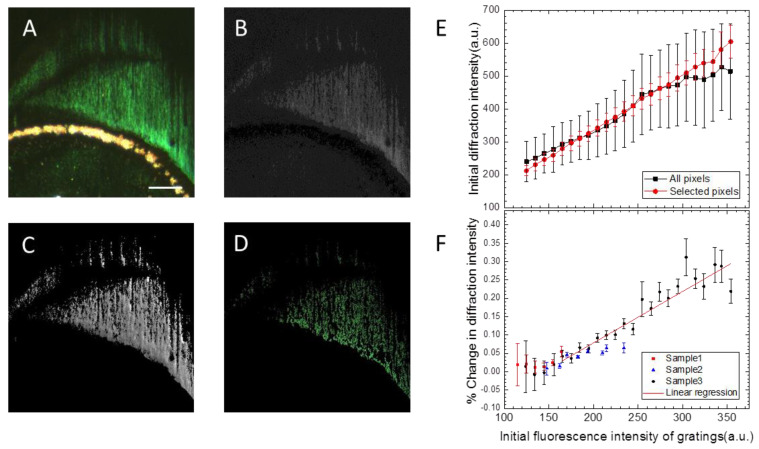
Image analysis reveals a stronger signal in pixels with color ratios consistent with Bragg diffraction and gratings with larger aspect ratios. (**A**) The initial diffraction image of a heterogeneous lipid multilayer grating. (**B**) The result of dividing the green-channel image of (**A**) by its red-channel image. As a result, the region of interest of the green diffraction intensity is identified. (**C**) The image of the green channel with the region of interest from (**B**) is divided by its fluorescent image. This ratio is used as a criterion for selecting pixels. (**D**) The final area is selected if it is within one standard deviation of the average green:red ratio from (**C**). (**E**) The graph shows the diffraction intensity as a function of its initial fluorescence intensity between selected pixels and all available pixels, indicating that grating lines with a larger height show a larger initial diffraction intensity. (**F**) Plots of the percentage of change in diffraction intensity response 5 min after the addition of thrombin among 3 different samples. This indicates that the signal is stronger in gratings with a larger height.

Figure 5A shows a diffraction image of a grating showing such heterogeneities. Some pixels are green, indicating Bragg diffraction, while others are not and indicate light scattered from the sample by other mechanisms. We first sought to identify only the pixels resulting from Bragg diffraction. We did this by separating an RGB color image into its individual grayscale channels. A pixel that represents Bragg diffraction should have a large signal in the green channel relative to the blue and red channels. We therefore divided the green channel by the red channel, to produce the grey scale image shown in Figure 5B. The bright pixels in this image represent the pixels that are the most green, or that have the highest green:red ratios. We set a threshold value and created a pixel map, so that only the pixels with a minimum green:red ratio were identified (Figure 5C).

We previously observed that grating diffraction efficiency depended linearly on grating thickness or height, up to a maximum grating height of about 1/10th of the line width [25]. We have also demonstrated that the multilayer thickness, or the height of the fluorescently labeled lipid nanostructures, is proportional to their fluorescence intensity [61]. Here, we therefore used fluorescence intensity as a relative measure of grating height. In order to test the hypothesis that gratings with a larger height show a stronger response, we further selected the most ideal pixels that showed a linear relationship between grating fluorescence intensity (or height) and diffraction intensity. We identified these optimal pixels by plotting the previously identified green pixels v/s fluorescence intensity of the same pixel. We then selected the pixels that were within one standard deviation of the mean diffraction intensity for that particular grating height or fluorescence intensity. This process led to the pixels shown in Figure 5D and the data plotted in Figure 5E. Analyzing the data this way allowed us to produce the graph shown in Figure 5F, which indicates that gratings with a larger height, as indicated by the relative fluorescence intensity, show a stronger response to the aptamer binding.

**Figure 6:**
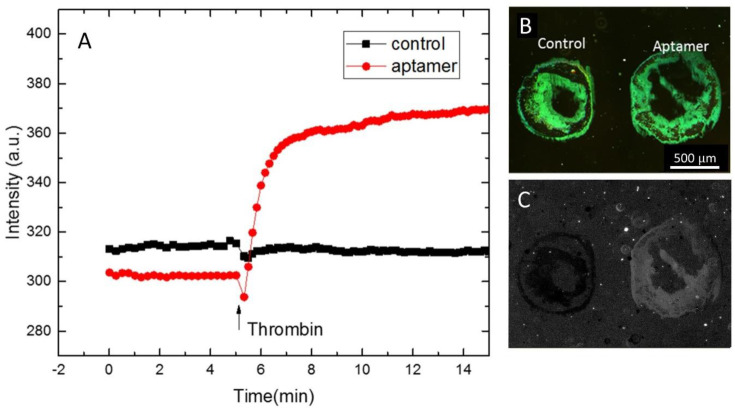
Detection of thrombin from optimized aptamer functionalized gratings. The lipid composition was DOPC:DOTAP 40:60, pixels with a maximum green/red ratio were chosen, and gratings were fabricated to have maximum heights as determined by the relative fluorescence intensity. (**A**) Sensorgram showing a change in diffraction from the aptamer functionalized gratings upon exposure to thrombin. (**B**) Initial diffraction intensity of lipid gratings printed onto PMMA. (**C**) Processed image after exposure to thrombin for 10 min. The green channel after exposure was divided by the green channel before exposure to show the change in diffraction intensity.

Using the knowledge gained about the optimal lipid composition, the importance of grating height, and the selection of pixels that represent Bragg diffraction, we were able to obtain high-quality sensorgrams such as the one shown in Figure 6A. Gratings containing DNA that did not contain the aptamer sequence were used as a negative control. The data were recorded at five frames per minute after incubation in the buffer for 30 min at ambient room temperature. The concentration of thrombin at 1 µM was introduced in the system after 5 min. The diffraction intensities from the videos were extracted using ImageJ. Figure 6B shows a diffraction image of the two gratings. The green intensity of the aptamer lipid grating increased by 20% after 10 min exposure to thrombin, while the control remained almost constant throughout the whole experiment. Figure 6C shows the green channel of the final image divided by the initial image, indicating a clear increase in the diffraction intensity of the analyte bound aptamer spot compared to the control. Compared to previous sensorgrams, the pixel selection process described here led to an improved signal to noise ratio.

## 4. Conclusions

The results indicate the suitability of the strategy described here for functionalizing lipid multilayer gratings with nucleic acid aptamers. The coupling of the analyte binding to nanostructural changes in the gratings leads to a detectable optical signal. The control of the lipid composition and grating nanostructure, particularly the grating height as qualitatively determined by the fluorescence measurement, was found to be crucial to obtain a robust signal from these transducers. The precise mechanism of transduction remains to be elucidated. The intensity of light diffracted from the gratings in this work was observed to increase upon analyte binding, which indicates that local structural changes are increasing the diffraction efficiency of the gratings. This increase could be caused by the presence of the protein analyte increasing grating height, local changes in the refractive index of the grating material, or changes in the shape induced by binding. It is currently unknown whether the size of the analyte plays a role in the signal, and the use of aptamers for smaller analytes such as ions and small molecules would help to narrow down the possibilities. Furthermore, the quantification of the binding affinity and specificity of the analyte to the aptamer functionalized gratings in this system could provide insights into the role of the lipid nanoenvironment on the aptamer function.

As a variety of aptamer sequences are available for the detection of different analytes, this platform is promising for integrating different sensor functionalities into the same surface multiplexed detection using microarray technology [44]. As each pixel in the camera can address a different area of the array, a highly parallel and effectively “wireless” readout of the different sensors in the array is possible. To illustrate this, if each individual pixel in a 1.4-megapixel CCD camera can be used to image a different sensor on an array formed over an area of <1 cm^2^, then 1.4 × 10^6^, different tests could be carried out on a single ~15 µL droplet. Further improvements in the lipid multilayer nanofabrication processes are expected to allow for improved sensitivity and limits of detection [67,68].
